# Light meal consumption among university students in China: A multi-regional cross-sectional study of behavioral patterns, influencing factors, and service optimization strategies under supply-side heterogeneity

**DOI:** 10.1371/journal.pone.0361891

**Published:** 2026-11-03

**Authors:** Hairu Zhang, Baoqi Zhou, Jiaxin Wang, Yue Li

**Affiliations:** 1 College of Economics and Management, Yanshan University, Qinhuangdao, China; 2 School of Artificial Intelligence (School of Software), Yanshan University, Qinhuangdao, China; 3 College of Economics and Management, Yanshan University, Qinhuangdao, China; 4 Hebei University of Chinese Medicine, Shijiazhuang, China; Lusofona University of Humanities and Technologies: Universidade Lusofona de Humanidades e Tecnologias, PORTUGAL

## Abstract

This study surveyed 270 college students from seven major geographic regions of China and, using multiple ordinal logistic regression, structural equation modeling (SEM), importance–performance analysis (IPA), and K-means clustering, examined their light meal consumption behavior, its determinants, and service optimization pathways. Results show that light meal consumption is characterized by diversified demands, regular-meal-oriented timing, rigid price sensitivity, and functionalized categories; specific dietary needs are the core driver of consumption frequency (B = −1.985, p < 0.001), whereas grade, gender, monthly living expenses, and campus supply conditions are not significant. In universities without a light meal supply, consumption intention follows an “attitude–norm/control–intention” multiple mediation pathway, with subjective norm showing the strongest direct effect (β = 0.392). In universities with a light meal supply, supply–demand gaps across all six service dimensions are highly significant (p < 0.001), with taste and variety falling into the IPA improvement quadrant as core shortcomings. K-means clustering identifies three student groups in supplied universities (high-satisfaction loyal, differentiated rational, and low-satisfaction wait-and-see) and two in non-supplied universities (high-demand expectant and low-demand wait-and-see). The findings confirm that intrinsic health demands, rather than external supply conditions, are the core driver; peer effects are a key lever; taste and variety are the primary bottlenecks to repeat purchases; and student heterogeneity necessitates stratified strategies.

## 1. Introduction

Against the backdrop of the Healthy China strategy, the nutritional and health status of young people has attracted significant attention. The *National Nutrition Plan (2017–2030)*, issued by the General Office of the State Council, explicitly calls for the development of healthy cafeterias and restaurants [1], while the “Year of Weight Management” campaign jointly launched by 16 departments including the National Health Commission has elevated the prevention and control of overweight and obesity to the level of a national initiative [2]. As the primary training grounds for young talent, universities must ensure that their food service not only guarantees food safety but also guides faculty and students toward healthy eating habits.

In recent years, light meals centered on the core concepts of low sugar, low fat, and high fiberhave quietly gained popularity in college cafeterias [3,4]. This new dining model emphasizes the authentic presentation of ingredients and minimally invasive cooking methods, aligning with contemporary college students’ pursuit of a healthy lifestyle [5]. Surveys show that over 80% of college students want to lose fat and weight, and nearly 60% have paid for light meals and other body-conscious products [6]. As digital natives, Generation Z’s health-conscious consumption is expanding from fat loss and body sculpting to daily wellness, and light meals are evolving from a niche demand into a mainstream trend. The*White Paper on Light Meal Consumption Behavior Among the Chinese Population*, released by the Chinese Nutrition Society, indicates that the penetration rate of light meal consumption among young people is rapidly increasing [7,8]. At the same time, the in-depth implementation of the “Weight Management Year” policy has further fueled the health consumption market [9]. Lightmeas, as a practical embodiment of the concept of integrating nutrition with health, is attracting increasing attention from consumers [10]. These trends indicate that the provision of light meals on college campuses is no longer merely about meeting students’ basic dietary needs but has become an important on-campus practice for implementing the Healthy China strategy [11].

However, existing research on light meal consumption on college campuses still has significant shortcomings. On the one hand, most domestic studies remain at the level of descriptive statistics; for example, surveys on demand for light meal outlets at specific universities have only presented students’ basic consumption intentions and price preferences [12,13], failing to deeply reveal the underlying mechanisms influencing consumption behavior. Some studies have proposed product design schemes for fat-reduction outlets but lack insights into specific demographic segments [14]. In addition, some scholars have explored pathways for optimizing light meals on college campuses from a supply-side perspective [15], conducted research and analysis on the business models of campus light meal restaurants [16], and examined the feasibility of promoting New Chinese-stylelight meals among young people [17]; Feng Xiaoyi et al. conducted a systematic analysis of the potential, advantages, and strategies for developing light meal catering in universities [18], while other scholars conducted demand surveys on the light meal markets in universities across different regions [19]. Some studies have also revealed, from a cognitive perspective, the gap between young consumers’ stereotypes of light meals and their actual expectations [20]. Additionally, the feasibility of introducing light meal options into university cafeterias has been explored from a logistics management perspective [21]. The aforementioned studies provide an important research foundation for this paper; however, most consist of descriptive analyses limited to a single university or region, and there remains a lack of comprehensive, nationwide, cross-institutional empirical research. Although international research on healthy dining in higher education institutions has attempted to broaden its perspective,such as studies on campus cafés revealing the disconnect between health preferences and consumption behavior [22], and research on the perception of oil and salt in Chinese-style cafeterias that developed quantitative assessment tools [23],such studies still primarily rely on descriptive statistics and single-indicator analysis. Finally, although some studies have attempted to incorporate the Theory of Planned Behavior to explore the intention to consume healthy foods [24,25], research that focuses on the specific population of college students and distinguishes differences in campus food supply remains scarce. In recent years, international scholars have conducted a large number of empirical studies on college students’ healthy eating behaviors.Vishwakarma et al. developed a model of college students’ food choices based on the Theory of Planned Behavior (TPB) [26]. Malara et al. found significant gender differences in college students’ dietary behaviors, with female students tending to make healthier food choices than male students [27]. Eryucel et al. extended the TPB framework to the realm of sustainable dietary behaviors and validated the important role of perceived behavioral control in college students’ healthy eating [28]. Silva et al. conducted a systematic review of strategies to improve adherence to healthy eating in college food service programs [29]. For colleges that have not yet established light-meal restaurants, how can they precisely align their supply arrangements with student needs? For those that have already established such facilities, in what areas should they optimize and upgrade their existing services? These two core questions urgently require answers through systematic empirical research.

To address the aforementioned research gaps, this study employs a questionnaire survey method and comprehensively utilizes various quantitative methods, including the proportional odds ordinal logit model, structural equation modeling, importance–performance analysis, and K-means cluster analysis. The study aims to:(1) identify the key factors influencing the frequency of students’ light meal consumption; (2) to explore the mechanisms underlying the formation of consumption intentions among students at universities without light meal offerings; (3) to evaluate the service quality and the degree of supply-demand alignment at universities that do offer light meals; (4) to segment the student market and identify the group characteristics of college students’ light meal consumption. Based on the above findings, this paper provides actionable strategic recommendations for the targeted planning and refined optimization of light meal services in Chinese university cafeterias.

## 2. Theoretical background

The Theory of Planned Behavior is a classic theoretical framework in social psychology for explaining and predicting human behavior. This theory posits that behavioral intention is the most direct factor influencing the occurrence of behavior, and that behavioral intention, in turn, is influenced by three core variables: behavioral attitude, which refers to an individual’s positive or negative evaluation of performing a specific behavior; subjective norm, which refers to the social pressure an individual perceives from significant others or groups; and perceived behavioral control, which refers to an individual’s perception of the ease or difficulty of performing the behavior [25].

The TPB has been widely applied in research on health food consumption. Studies on health foods have found that consumers’ health awareness influences purchase intention through the sequential mediation of attitudes and desires [24]. Other studies have indicated that willingness to pay and price are the strongest predictors of health food purchase intention, with effects that even surpass those of attitudes and health awareness [13]. However, most of these studies have focused on the general consumer population, and research targeting college students,who are price-sensitive and heavily influenced by their peers,remains relatively scarce.

## 3. Methodology

### 3.1. Ethical statement

This study adheres to the ethical guidelines of the American Psychological Association, and the research protocol has been approved by the Ethics Committee of Hebei University of Chinese Medicine (Approval No.: YXLL202601013). Before completing the questionnaire, all participants were informed of the study’s purpose, the principle of anonymity, and the voluntary nature of participation through the questionnaire’s introduction, and they began answering only after clicking to give their consent online. As all participants were adult college students who voluntarily completed the questionnaire, no separate informed consent was obtained from their guardians.

### 3.2. Participants

This study surveyed full-time undergraduate students at colleges and universities across China. A combination of quota sampling and convenience sampling was employed, with China’s seven major geographic regions serving as the quota dimensions. Sample sizes were allocated with reference to the number of institutions and student populations in each region. Questionnaires were distributed online via QR codes disseminated through campus social media groups and offline campus activities. The regional distribution of participant universities is shown in Table 1. A total of 303 questionnaires were distributed, and 300 were returned, yielding a response rate of 99.0%. After excluding 30 invalid responses due to patterned answering or missing key items, 270 valid questionnaires were retained, resulting in an effective response rate of 90.0%.

**Table 1:** Regional distribution of participant universities.

Geographic Region	Provinces	Sample Size	Percentage (%)
East China	Shanghai, Jiangsu, Zhejiang, Anhui, Fujian, Jiangxi, Shandong	84	31.1%
North China	Beijing, Tianjin, Hebei, Shanxi, Inner Mongolia	46	17.4%
Central China	Henan, Hubei, Hunan	32	11.9%
Southwest China	Chongqing, Sichuan, Guizhou, Yunnan, Xizang	32	11.9%
South China	Guangdong, Guangxi, Hainan	26	9.6%
Northeast China	Liaoning, Jilin, Heilongjiang	30	11.1%
Northwest China	Shaanxi, Gansu, Qinghai, Ningxia, Xinjiang	20	7.4%
Total		270	100%

It should be noted that, because this study aims to explore in depth the mechanisms underlying consumption behavior in the context of light meals in university canteens rather than to provide a national point estimate, a combination of quota sampling and convenience sampling was adopted. Although this limits the unconditional generalizability of the study’s conclusions to the entire population of Chinese university students, the strict adherence to geographic quotas across the seven major regions ensured basic coverage of the sample across different areas and allowed for the effective capture of common patterns among universities in different regions. In addition, the relatively balanced distribution of the sample with respect to gender, grade, and monthly living expense range provided sufficient statistical power for examining the internal associations among the variables in this study.

### 3.3. Measurement instruments

The research team designed a structured questionnaire tailored to the study’s objectives. It consisted of 19 questions covering five major modules: demographic information, light meal consumption behaviors and preferences, the TPB scale, the IPA evaluation scale, and open-ended comments. Both the TPB scale and the IPA scale used a five-point Likert scale (1 = “Strongly Disagree,” 5 = “Strongly Agree”). A small-scale pilot survey was conducted before the questionnaire was officially distributed, and the wording of some items was optimized based on the feedback received

### 3.4. Analytical strategy

Data analysis was conducted using SPSS 26.0 and AMOS 24.0 software, following these specific steps:

(1) Data Cleaning and Reliability/Validity Testing: Invalid questionnaires with response times shorter than 60 seconds or showing obvious patterns of answering were excluded. Cronbach’s α was used to test questionnaire reliability, while the KMO test and Bartlett’s sphericity test were used to assess questionnaire validity. Additionally, Harman’s single-factor test was conducted to evaluate common-method bias.
(2) Descriptive Statistics and Randomness Tests: Descriptive statistical analysis was performed on the sample’s demographic characteristics and consumption behavior, and the randomness of the sample for key variables was verified using the run test. Descriptive statistics is the most fundamental quantitative analysis method in statistics; its core purpose is to objectively and systematically quantify and visualize the basic data characteristics of the survey sample without making complex causal inferences, but rather to reveal the distribution patterns and fundamental characteristics of the data itself. The run-test is used to determine whether a sequence of sample observations is randomly distributed. If the asymptotic significance P-value of the test is greater than 0.05, it indicates that the sample data do not exhibit systematic patterns, the sampling process satisfies the randomness assumption, and the data can be further used for parametric tests and regression analysis.
(3) Analysis of Factors Influencing Consumption Frequency: With the frequency of light meal consumption as the dependent variable, a proportional odds ordinal logit model model was constructed to identify its core driving factors and constraining factors. The proportional odds ordinal logit model is specifically designed for regression analysis where the dependent variable is an ordered multi-class variable. Its core function is to use maximum likelihood estimation to quantify the direction, strength, and statistical significance of the independent variables’ effects on changes in the ordinal levels of the dependent variable, thereby identifying the core driving factors.
(4) Analysis of the Mechanism of Consumption Intentions (Universities Without Light Meal Offerings): Structural Equation Modeling was employed to test research hypotheses within the framework of the Theory of Planned Behavior. Structural Equation Modeling is a multivariate statistical method that integrates factor analysis and path analysis. It can simultaneously test complex causal relationships among multiple variables, is suitable for analyzing latent variables such as attitudes and norms that cannot be directly observed, and can decompose the effects between variables into direct and indirect effects. It is an effective tool for testing theoretical models and mediating mechanisms.
(5) Service Quality Assessment (for universities offering light meals): An importance–performance analysis model was constructed, with importance as the horizontal axis and satisfaction as the vertical axis, to plot a four-quadrant matrix and identify the strengths, maintenance, opportunity, and improvement areas of the light meal service.
(6) Market Segmentation: K-means clustering analysis was conducted separately for the two groups of college students with and without a light meal supply, using key variables as clustering factors, and principal component analysis was employed to reduce the dimensionality of the clustering results for visualization. K-means clustering is the most classic partitioning clustering algorithm in unsupervised machine learning. Its core objective is to automatically partition n samples into a preset number of K clusters based on feature similarity, ultimately achieving maximum similarity within clusters and maximum diversity between clusters, thereby completing the unsupervised grouping and profiling of the samples.

## 4. Research findings

### 4.1. Reliability and validity tests

The results of the reliability and validity tests showed that the Cronbach’s α coefficient for the total scale was 0.838, the KMO value was 0.81, and the p-value for Bartlett’s sphericity test was < 0.001, indicating that the questionnaire data possess good reliability and validity. Harman’s single-factor test revealed that the first common factor explained 28.6% of the variance (<40%), indicating that there was no serious common method bias, as shown in Table 2.

**Table 2:** Cronbach’s alpha values and Bart Sphericity Test.

Cronbach^’^s Alpha	KMO	Bart Sphericity Test (p)
0.838	0.811	p < 0.001

### 4.2. Factor analysis

To further validate the structural validity of the scale items, an exploratory factor analysis using principal component analysis was conducted on scale items Q9–Q12, followed by factor rotation using the Kaiser-Mertin normalization method.

In accordance with the four-dimension framework proposed by the Theory of Planned Behavior (TPB), this factor analysis forced the extraction of four common factors. The results showed that the cumulative variance explained reached 69.296%, exceeding the statistical threshold of 60%. This indicates that the four common factors, extracted under theoretical guidance, effectively capture the core information contained in the items. The factor extraction yielded ideal results, and the structural information of the scale was fully explored.

### 4.3. Randomness test

This study conducted a runs test on three key variables, grade level, monthly living expense range, and expected purchase frequency of light meals, to verify the randomness of the sample data. The results are shown in Table 3. The asymptotic significance (two-tailed) p-values for the three variables were 0.885, 0.908, and 0.216, respectively, all of which were greater than 0.05. This indicates that each sample sequence is randomly distributed, that there is no systematic clustering bias in the sampling process, and that the data exhibit good randomness, thereby satisfying the basic prerequisites for the proportional odds ordinal logit regression and structural equation modeling analyses.

**Table 3:** Results of runs tests for key variables.

Item	Grade Level	Monthly Living Expense Range	Expected Frequency of Light Meal Purchase
Median	3	3	4
Number of Cases < Median	93	112	123
Number of Cases ≥ Median	177	158	147
Total Number of Cases	270	270	270
Number of Runs	124	133	145
Z	0.144	.115	1.237
Asymptotic Significance (two-tailed)	0.885	.908	0.216

### 4.4. Descriptive statistics on college students’ demand and preferences for light meal consumption

The survey found that 59.8% of students have specific dietary needs—such as weight loss, stomach health, and low-sugar diets—with most having multiple needs. The proportion of students with the single need of “weight loss only” was 22.7%, indicating that students’ health concerns have moved beyond simple fat loss and now reflect a combination of multiple health priorities. A summary of the key data across various dimensions is shown in Table 4.

**Table 4:** Summary of core indicators of light meal consumption demand and preferences.

Dimension	Category / Range	Percentage (%)
Specific dietary needs	Having specific dietary needs	59.8%
	Weight loss only	22.7%
Consumption period	Lunch	72.3%
	Dinner	65.1%
	Breakfast	28.5%
	Late-night snack	9.2%
Price acceptability	6–10 RMB	35.2%
	11–15 RMB	27.3%
	Above 15 RMB	8.0%
Food type preference	Stomach-soothing meals	43.8%
	Fat-reduction meals	39.5%
	Low-sugar pastries	27.4%
	Multigrain rice	24.7%

Generation Z’s consumption of light meals exhibits four key characteristics: diversified demand, meal-time consumption, price rigidity, and functional product categories, providing a demand-side basis for future supply optimization.

### 4.5. Factors influencing the consumption frequency of light meals among college students

“The expected frequency of purchasing light meals” is an ordinal multi-category variable comprising four levels. It was therefore used as the dependent variable, with grade, gender, monthly living expenses, specific dietary needs, and whether a light meal canteen is available on campus as independent variables. Gender and grade served as demographic control variables to control for inherent differences in dietary behavior across groups; such variables are conventional controls in logistic regression studies of campus dietary behavior [30]. Monthly living expenses represent students’ disposable spending capacity and reflect individual payment constraints on premium healthy foods such as light meals [30]. Specific dietary needs correspond to individuals’ subjective dietary motivations; previous studies have shown that intrinsic demands such as fat reduction and specific dietary restrictions significantly increase the probability of choosing healthy food services. According to the industry survey in the 2020 White Paper on Light Meal Consumption Behavior of the Chinese Population, the primary driver for young people aged 19–45 to choose light meals is subjective dietary needs related to weight loss and health management. Whether a light meal canteen is available on campus reflects the objective availability of healthy food services on campus; drawing on food environment theory, external supply conditions constrain individuals’ dietary choice sets, and this environmental variable was therefore included by referring to the research approach of campus food environment studies. The variable coding and assignment are shown in Table 5.

**Table 5:** Variable coding and assignment for the ordinal logistic regression model.

Variable Type	Variable Name	Variable Assignment
Dependent variable	Expected frequency of purchasing light health meals	Once a day = 1; 3–5 times per week = 2; 1–2 times per week = 3; Occasionally = 4
Independent variable	Grade level	Freshman = 1; Sophomore = 2; Junior = 3; Senior = 4; Postgraduate = 5
Independent variable	Gender	Male = 0; Female = 1
Independent variable	Monthly living expense	<1000 RMB = 1; 1000–1500 RMB = 2; 1500–2000 RMB = 3;> 2000 RMB = 4
Independent variable	Specific dietary needs	No = 0; Yes = 1
Independent variable	Availability of light meal canteen on campus	Yes = 1; No = 2

**(1) Parallel lines test**

The parallel lines test is used to verify the null hypothesis that “the regression coefficients of the independent variable are equal across the categorical levels of the dependent variable.” The test results are shown in Table 6: χ² = 19.062, df = 20, p = 0.518 > 0.05. This indicates that, based on the parallel lines test, there are no significant differences in the regression coefficients across the categories of the dependent variable; the model is appropriately specified and can be used for subsequent analysis.

**Table 6:** Test of parallel lines for the ordinal logistic regression model.

Model	−2 Log Likelihood	Chi-Square	df	Sig.
Null Hypothesis	350.266			
General	331.204	19.062	20	0.518

**(2) Likelihood ratio test**

The likelihood ratio test was used to compare the goodness of fit of nested models. As shown in Table 7, the −2 log-likelihood of the final model decreased from 412.520 under the null hypothesis to 350.266, with a chi-square value of 62.254 (df = 10), p < 0.001, indicating that the overall model is significant and that the independent variables jointly explain the variation in the dependent variable effectively.

**Table 7:** Likelihood ratio test for the ordinal logistic regression model.

Model	−2 Log Likelihood	Chi-Square	df	Sig.
Null Hypothesis	412.520			
General	350.266	62.254	10	0.000

**(3) Parameter estimation results**

The parameter estimation results are shown in Table 8. Among all independent variables, the presence of specific dietary needs significantly affected the expected purchase frequency of light meals. In the proportional odds ordinal logit model, a negative regression coefficient B indicates that students with specific dietary needs had a significantly increased cumulative log-odds of shifting toward a higher consumption frequency level. Grade, gender, monthly living expense range, and whether the university has a light meal canteen showed no statistically significant effects (p > 0.05).

**Table 8:** Ordinal logistic regression results for factors influencing light meal consumption frequency.

Variable	B	Std. Error	Wald	Sig.	95% CI Lower	95% CI Upper	OR (95% CI)
[Q1=1 Freshman]	0.753	0.634	1.410	0.235	−0.490	1.997	2.123 (0.613, 7.367)
[Q1=2 Sophomore]	−0.152	0.476	0.102	0.749	−1.085	0.781	0.859 (0.338, 2.184)
[Q1=3 Junior]	−0.222	0.487	0.208	0.648	−1.177	0.733	0.801 (0.308, 2.081)
[Q1=4 Senior]	−0.397	0.482	0.679	0.410	−1.343	0.548	0.672 (0.261, 1.730)
[Q2=1 Male]	−0.005	0.274	0.000	0.986	−0.542	0.532	0.995 (0.582, 1.702)
[Q3=1 Monthly expense < 1000]	−0.521	0.577	0.817	0.366	−1.652	0.609	0.594 (0.192, 1.839)
[Q3=2 Monthly expense 1000-1500]	0.554	0.363	2.337	0.126	−0.156	1.265	1.740 (0.856, 3.543)
[Q3=3 Monthly expense 1500-2000]	0.669	0.361	3.437	0.064	−0.038	1.375	1.952 (0.963, 3.955)
[Q4=1 With specific dietary needs]	−1.985	0.281	50.017	<0.001	−2.535	−1.435	0.137 (0.079, 0.238)
[Q7=1 Light-meal canteen available]	−0.121	0.250	0.233	0.629	−0.610	0.369	0.886 (0.543, 1.446)

The above results indicate that specific dietary needs are the core factor driving college students to increase their expected consumption frequency of light meals. When students have subjective dietary demands such as fat reduction or stomach care, their likelihood of choosing a higher consumption frequency level increases markedly. In contrast, demographic characteristics, disposable living expenses, and campus light meal supply conditions did not exert an overall statistically significant effect on consumption frequency level in this sample. The p-value for the monthly living expense group of 1,500–2,000 RMB was 0.064, which is marginally close to significance but does not reach the α = 0.05 threshold, and is therefore insufficient to support the conclusion that “living expenses as a whole alter consumption frequency level.” This also indirectly suggests that students’ willingness to consume light meals derives more from their own intrinsic motivation for healthy eating rather than being constrained solely by external conditions such as the level of living expenses or whether the university has a light meal canteen.

### 4.6. Mechanism of consumption intention: an analysis based on students from universities without light meal supply

(1) Model construction

For universities without a light meal canteen, a total of 126 valid questionnaires were collected. According to the empirical sample size guideline proposed by Bentler and Chou, the sample size should be 5–10 times the number of observed items. This study included 11 observed items in total; the 10-times criterion corresponds to a sample size of 110, and the 126 valid responses in this study meet this empirical standard. Based on the theory of planned behavior (TPB) and combined with the specific context of light meal consumption in universities, a structural equation model was constructed, as shown in Fig 1, to analyze the formation mechanism of students’ light meal consumption intention.The four constructs are defined as follows:

**Fig 1:**
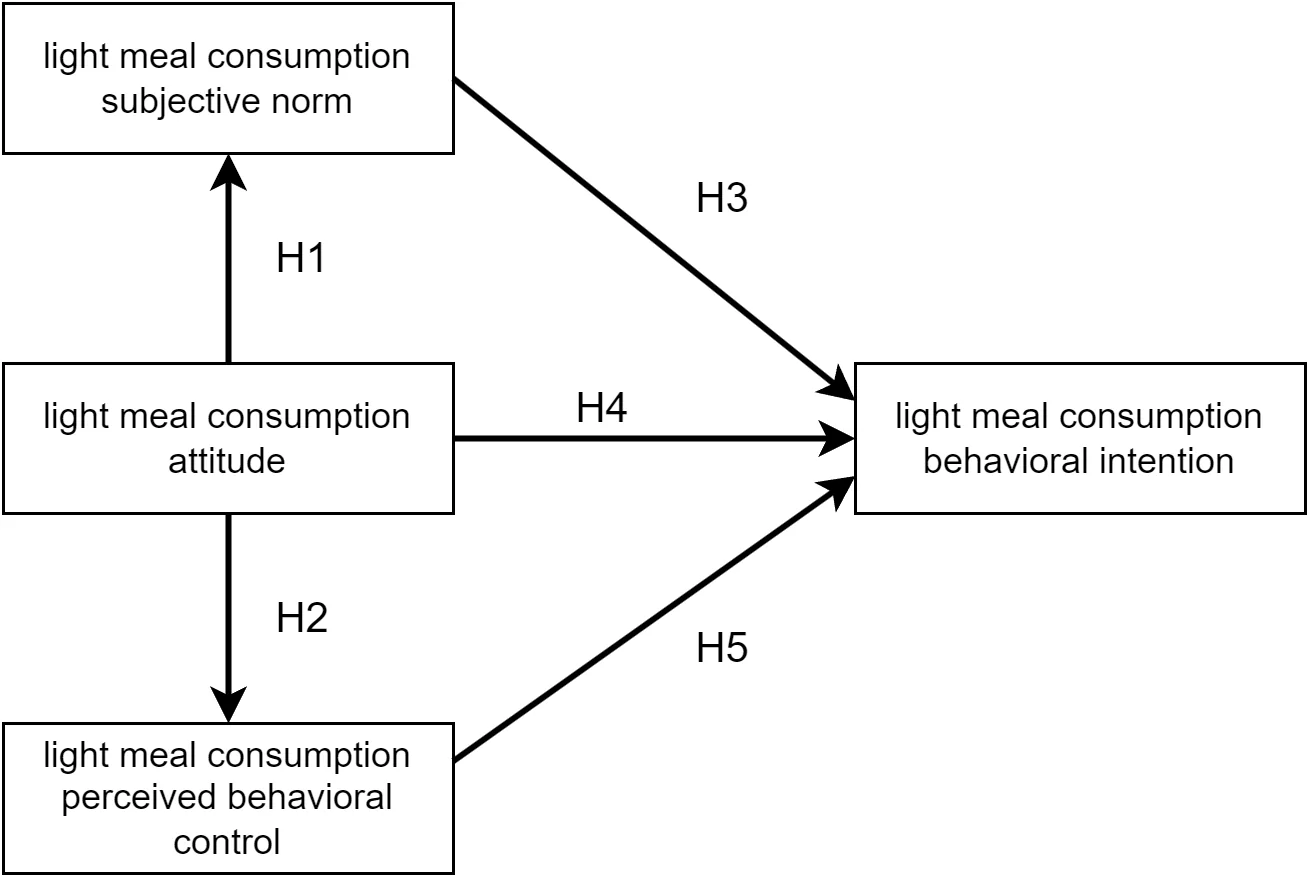
Light Meal Consumption Intention Model.

Light Meal Consumption attitude: College students’ subjective evaluations of purchasing and consuming light meals at the campus cafeteria, including positive or negative emotional tendencies regarding health benefits and taste expectations.

Light Meal Consumption subjective norm: The influence on college students’ decision to purchase light meals stemming from perceived pressure from significant others or social groups,such as the prevalence of light meal consumption among peers or the emergence of healthy eating as a campus trend.

Light Meal Consumption perceived behavioral control: The ease or difficulty university students perceive in carrying out light meal purchase behaviors, based on factors such as perceived convenience of purchase, product reliability, and information transparency within the cafeteria.

Light Meal consumption behavioral intention: The strength of university students’ subjective willingness to actively try, regularly purchase, or recommend light meals from the campus cafeteria to others in the near future.

Based on this, the following hypotheses are proposed:

Hypothesis 1: Light Meal Consumption attitude has a positive influence on Light Meal Consumption subjective norm.

Hypothesis 2: Light Meal Consumption attitude has a positive effect on Light Meal Consumption perceived behavioral control.

Hypothesis 3: Light Meal Consumption subjective norm has a positive effect on Light Meals consumption behavioral intention.

Hypothesis 4: Light Meal Consumption attitude has a positive effect on Light Meals consumption behavioral intention.

Hypothesis 5: Light Meal Consumption perceived behavioral control has a positive effect on Light Meal Consumption perceived behavioral control

Hypothesis 6: Light Meal Consumption subjective norm mediates the relationship between Light Meal Consumption attitude and Light Meals consumption behavioral intention.

Hypothesis 7: Light Meal Consumption perceived behavioral control mediates the relationship between Light Meal Consumption attitude and Light Meals consumption behavioral intention.

**(2) Model Fit Test**

The model fit results are shown in Table 9. χ²/df = 1.319 (<3), GFI = 0.936 (>0.90), RMSEA = 0.050 (<0.08), IFI = 0.972 (>0.95), TLI = 0.960 (>0.95), CFI = 0.971 (>0.95). All core fit indices meet or exceed the ideal standards, indicating that the structural equation model fits the data very well, is reasonably specified, and effectively explains the relationships among the variables.

**Table 9:** Fit indices for the structural equation model.

Index	Model Fit Value	Criterion / Threshold
CMIN/DF	1.319	1 < CMIN/DF < 3
GFI	0.936	>0.90
RMSEA	0.050	<0.08
IFI	0.972	>0.95
TLI	0.960	>0.95
CFI	0.971	>0.95

The composite reliability (CR) and average variance extracted (AVE) results of the measurement model are shown in Table 10. The CR values of light meal consumption subjective norm and light meal consumption perceived behavioral control were both greater than 0.7, and their AVE values were greater than 0.5, indicating that these two constructs have good reliability and convergent validity. The CR of light meal consumption behavioral attitude was 0.643, slightly below the threshold of 0.7, and its AVE was 0.487, slightly below 0.5; the CR of light meal consumption behavioral intention reached 0.774, but its AVE was 0.476, falling below 0.5, mainly due to the low standardized factor loading (0.391) of item YX4. According to related studies, when CR meets the requirement, an AVE slightly below 0.5 remains acceptable; therefore, the convergent validity of all constructs in this study is generally at an acceptable level.

**Table 10:** Construct reliability and convergent validity.

Latent Variable	Number of Items k	CR	AVE
Light Meal Consumption Attitude	2	0.643	0.487
Light Meal Consumption Subjective Norm	2	0.792	0.665
Light Meal Consumption Perceived Behavioral Control	3	0.749	0.502
Light Meal Consumption Behavioral Intention	4	0.774	0.476

The Fornell–Larcker criterion was then used to examine the discriminant validity of the latent variables, and the results are shown in Table 11. The diagonal values in the table are the square roots of the AVE of each construct, and the lower triangle contains the correlations among the latent variables. The results show that the square root of the AVE of each construct was greater than its correlations with the other constructs, satisfying the Fornell–Larcker criterion, which indicates that the four latent variables in this study have good discriminant validity. In addition, a 5,000-replication bootstrap test was conducted; the 95% bias-corrected confidence intervals of the correlations among the latent variables all excluded zero, indicating that the correlations among the latent variables were all significant.

**Table 11:** Results of discriminant validity.

	Light Meal Consumption Attitude	Light Meal Consumption Subjective Norm	Light Meal Consumption Perceived Behavioral Control	Light Meal Consumption Behavioral Intention
Light Meal Consumption Attitude	0.698			
Light Meal Consumption Subjective Norm	0.506	0.815		
Light Meal Consumption Perceived Behavioral Control	0.450	0.372	0.709	
Light Meal Consumption Behavioral Intention	0.623	0.372	0.600	0.690

(3) Results Analysis

Path analysis and standardization effect analysis, as shown in Fig 2, indicated that all hypotheses were supported. Light meal consumption attitude has a significant positive effect on light meal consumption subjective norm (β = 0.52, p < 0.001) and a significant positive effect on light meal consumption perceived behavioral control (β = 0.48, p < 0.001); Hypotheses H1 and H2 are supported. Light meal consumption subjective norm has a significant positive effect on light meal consumption behavioral intention (β = 0.46, p < 0.001); Hypothesis H3 is supported. Light meal consumption attitude has a significant positive effect on light meal consumption behavioral intention (β = 0.31, p < 0.05), confirming Hypothesis H4. Light meal consumption perceived behavioral control has a significant positive effect on light meal consumption behavioral intention (β = 0.31, p < 0.01), confirming Hypothesis H5. Among these, light meal consumption subjective norm has the strongest direct effect on light meal consumption behavioral intention (β = 0.46, p < 0.001).

**Fig 2:**
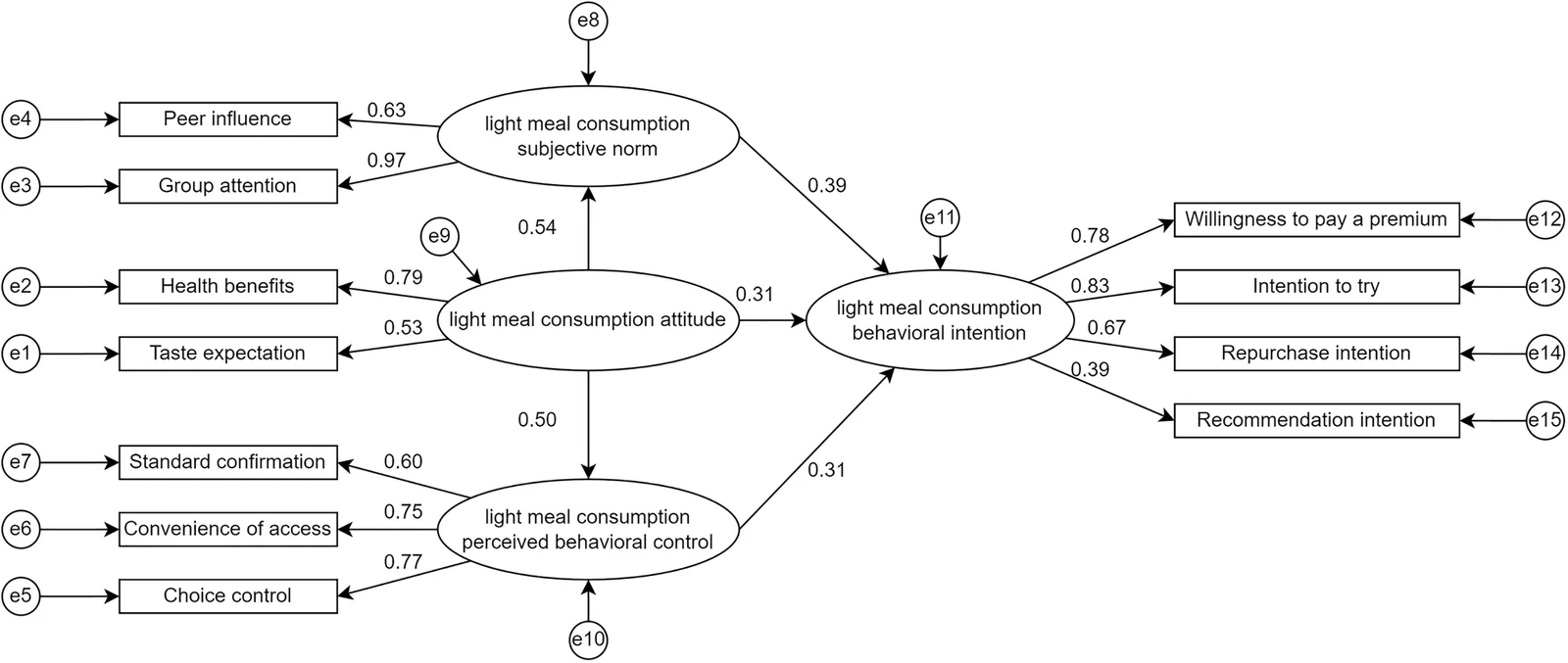
SEM Path Analysis of Light Meal Intention.

The results of the direct effect analysis are shown in Table 12, and the results of the standardized effect analysis are shown in Table 13.

**Table 12:** Hypothesis testing results for direct effects.

Hypothesis	Path Relationship	Standardized Coefficient	C.R.	P
H1	Behavioral attitude → Subjective norm	0.52	3.980	<0.001
H2	Behavioral attitude → Perceived behavioral control	0.48	3.431	<0.001
H3	Subjective norm → Behavioral intention	0.392	2.968	0.003
H4	Behavioral attitude → Behavioral intention	0.310	1.985	0.047
H5	Perceived behavioral control → Behavioral intention	0.307	2.696	0.007

**Table 13:** Standardized effects decomposition.

Effect Path	Direct Effect	Indirect Effect	Total Effect	Proportion (%)	P
Behavioral attitude → Behavioral intention	0.310	0.366	0.676	45.86%	0.047
Behavioral attitude → Subjective norm → Behavioral intention	-	0.212	-	31.36%	0.000
Behavioral attitude → Perceived behavioral control → Behavioral intention	-	0.154	-	22.78%	0.002
Subjective norm → Behavioral intention	0.392	-	0.392	-	0.003
Perceived behavioral control → Behavioral intention	0.307	-	0.307	-	0.007

First, light meal consumption attitude has both direct and indirect influences on light meal consumption behavioral intention.Light meal consumption attitude not only directly influence light meal consumption behavioral intention (β = 0.310) but also exerts indirect effects through two pathways: from attitude to subjective norm to intention” (indirect effect = 0.212) and from attitude to perceived behavioral control to intention” (indirect effect = 0.154).The sum of these indirect effects (0.366) exceeds the direct effect (0.310). This indicates that students’ positive evaluations of the health value and taste of light meals further amplifies their consumption intentions by strengthening perceived social norms and perceived feasibility, and that attitude influences consumption decisions through multiple pathways. Second, light meal consumption subjective norm has the strongest direct influence. The standardization coefficient (β = 0.392) for the relationship between light meal consumption subjective norm and light meal consumption behavioral intention is the highest among all direct pathways, confirming the significant peer effect among college students: the healthy consumption choices of classmates and friends have a far greater influence on individual decision-making than personal attitudes and perceived feasibility. This finding aligns closely with the characteristics of college students who are highly social and easily influenced by group identity, and provides empirical support for word-of-mouth strategies in promoting light meals on campus.

Third, light meal consumption perceived behavioral control also plays a key role. The influence of light meal consumption perceived behavioral control on light meal consumption behavioral intention (β = 0.307) is primarily reflected at perceived feasibility: the more positively students perceive aspects such as whether the cafeteria, such as whether cafeterias strictly adhere to low-oil and low-sugar standards, whether purchasing is convenient, and whether students can easily find suitable options while managing their weight, the stronger their consumption intentions. This indicates that even if students have a positive attitude toward light meals and are influenced by those around them, their willingness to consume will still be suppressed if the purchasing process is cumbersome or if they have doubts about product quality, highlighting the importance of building convenience and trust on the supply side.

### 4.7. Service quality assessment: an analysis based on students from universities with light meal supply

To identify service shortcomings and areas for improvement in colleges with light meal canteens., this section employs the Importance-Performance Analysis (IPA) model to evaluate six core dimensions of light meal services from the students’ perspective.

**(1) Model construction and evaluation indicators**

The IPA model constructs a four-quadrant matrix by measuring consumers’ perceptions of the importance of service attributes and their satisfaction ratings, thereby identifying service quality shortcomings and guiding the prioritization of resource allocation. Its core theoretical foundation is the Expectation-Difference paradigm, according to which consumer satisfaction stems from the gap between expectations (importance) and actual perceptions.

This study developed an evaluation indicator system, as shown in Table 14, covering six dimensions of light meal offerings in university cafeterias, simultaneously collecting importance ratings and satisfaction ratings.

**Table 14:** Evaluation indicators for light meal supply in university canteens.

No.	Evaluation Factor
1	Taste and flavor of the dishes
2	Freshness and quality of ingredients
3	Variety of dish types
4	Price and value for money
5	Clear labeling of nutritional information
6	Convenience of purchase and waiting time

**(2) Significance Test**

To verify the statistical significance of the supply-demand gaps across each dimension, a paired-sample t-test was conducted on the importance and satisfaction scores for the six dimensions,as shown in Table 15. The results show that the mean differences for all dimensions were positive (0.472–0.736), and all p-values were less than 0.001, indicating that universities currently operating light-meal canteens exhibit a genuine and highly significant supply-demand mismatch across all dimensions of light-meal services, rather than a result of sampling error. Among these, the largest gap was observed in the variety of dishes (0.736), while the smallest gap was found in purchasing convenience and waiting time (0.472).

**Table 15:** Paired-sample t-test results for importance–satisfaction gaps.

Evaluation Dimension	Mean Difference (Importance − Satisfaction)	95% CI for Mean Difference	t	P
Variety of dish types	0.736	[0.547,0.925]	7.705	<0.001
Taste and flavor of the dishes	0.729	[0.565,0.894]	8.767	<0.001
Price and value for money	0.653	[0.447,0.858]	6.278	<0.001
Freshness and quality of ingredients	0.646	[0.464,0.828]	7.007	<0.001
Clear labeling of nutritional information	0.618	[0.423,0.813]	6.271	<0.001
Convenience of purchase and waiting time	0.472	[0.269,0.676]	4.591	<0.001

**(3) IPA matrix and analysis of results**

An IPA four-quadrant matrix was constructed with the overall mean importance score (4.19) on the vertical axis and the overall mean satisfaction score (3.55) on the horizontal axis, as shown in Table 16, Fig 3. The strategic implications of each dimension are as follows:

**Table 16:** IPA matrix results for light meal service quality.

Quadrant	Evaluation Factor	Strategic Implication
Strengths (High importance – High satisfaction)	Freshness and quality of ingredients	Core competitive advantage; continue to maintain
Stable Area (Low importance – High satisfaction)	Convenience of purchase and waiting time	Non-critical advantage; maintain current status
Opportunity Area (Low importance – Low satisfaction)	Price and value for money; Clear labeling of nutritional information	Potential areas for improvement; can serve as differentiation strategy
Areas for Improvment (High importance – Low satisfaction)	Taste and flavor of the dishes; Variety of dish types	Critical shortcomings; prioritize resource allocation for immediate improvement

**Fig 3:**
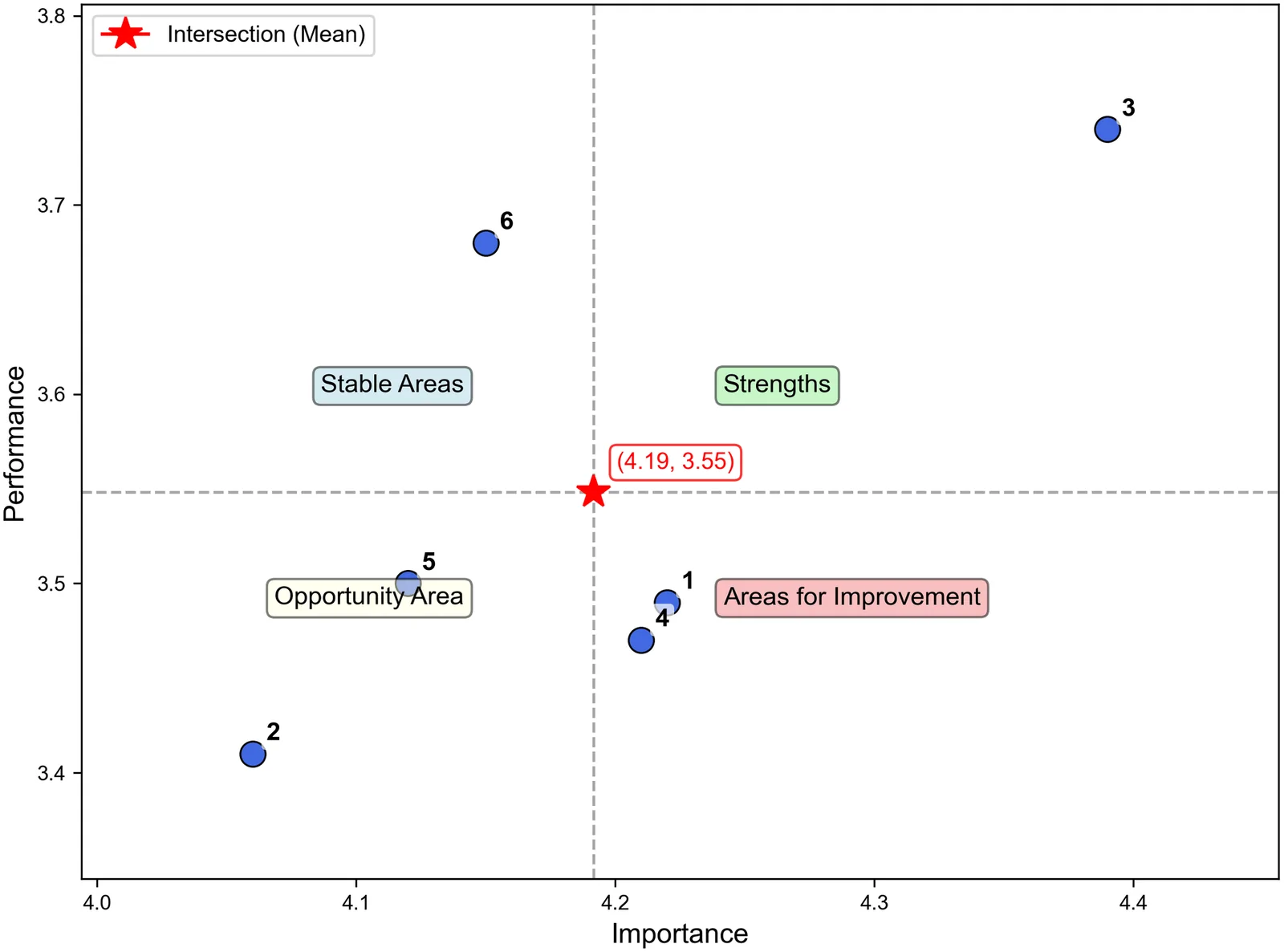
IPA Model Results for Light Meal Services.

Strengths: Freshness and quality of ingredients. Both the importance (4.39) and satisfaction (3.74) scores for this dimension are relatively high, aligning closely with the core positioning of light meals as healthy and natural.This represents the most compelling competitive advantage of current light meal services. Universities should continue to strengthen this advantage by consolidating student trust through measures such as displaying ingredient traceability information at service counters and showcasing fresh ingredients on-site.

Stable Areas: Convenience of purchase and wait times. The importance score of this dimension (4.15) was slightly lower than the overall mean importance, while its satisfaction score (3.68) was higher than the overall mean satisfaction, indicating that existing service processes already largely meet student needs. This is a nice-to-have advantage that does not require additional resource investment; efforts should instead be prioritized toward the Areas for Improvement.

Opportunity Area: The price and value for money of dishes, and clear labeling of nutritional information. The importance (4.06) and satisfaction (3.41) of dish price and value for money were both lower than the overall means. Students’ price sensitivity was not the highest, yet satisfaction was relatively low, suggesting that the problem lies in insufficient value perception rather than excessively high prices per se. The importance (4.12) and satisfaction (3.50) of clear labeling of nutritional information were likewise both lower than the overall means. Students have expectations in this area, but current labeling is unclear. Both metrics can be improved with minimal investment—for example, introducing combo meals to enhance the perceived value for money and listing calories and nutritional information on menus are low-cost, high-return optimization entry points.

Areas for Improvement: Texture and flavor of dishes, and variety of menu options. This is the most critical finding in the IPA model. The importance of texture and flavor (4.22) is the highest among all dimensions, yet satisfaction (3.49) remains low; “healthy but not tasty” is the primary bottleneck limiting consumption frequency and repurchase intent. The importance of variety (4.21) also ranks high, yet satisfaction (3.47) is severely lacking, failing to meet students’ needs in different scenarios such as weight loss, stomach care, and snack breaks. Both metrics show a significant gap between “high expectations and low fulfillment,” representing the core shortcomings that light meal services most urgently need to address by concentrating resources.

### 4.8. Market segmentation: Profiling the audience for light meals at universities using k-means clustering

To accurately identify the consumption characteristics of different student groups and achieve the goal of “moving away from a one-size-fits-all approach and implementing targeted operations,” this section uses the K-means clustering algorithm to segment students into two categories: those at universities with and without light meal canteens.

(1) Algorithm principles and selection of clustering factors

K-means clustering is an unsupervised machine learning method designed to automatically partition n samples into K clusters based on feature similarity, ensuring high similarity within clusters and maximum difference between clusters. Its core logic is as follows: K initial cluster centers are selected at random; the distance from each sample to the cluster centers is calculated, and the sample is assigned to the nearest cluster; the cluster centers are then iteratively updated until convergence is achieved.

For the group without a light meal canteen (n = 126), the scores of the four dimensions of the TPB scale, light meal consumption behavioral attitude, light meal consumption subjective norm, light meal consumption perceived behavioral control, and light meal consumption behavioral intention, were selected as clustering factors; for the group with a light meal canteen (n = 144), the satisfaction scores of the six IPA dimensions were selected as clustering factors. Before clustering, all continuous variables were standardized to eliminate the influence of dimensional units. Basic demographic information did not participate in the calculation of clustering distances and was used only after clustering was completed to analyze the sample characteristics of different user groups.

**(2) Selection of vthe K value**

The optimal K value was determined comprehensively by combining three evaluation indicators—the elbow method, the silhouette coefficient, and the Calinski–Harabasz (CH) index—supplemented by cluster sample balance and business interpretability, so as to avoid clustering bias caused by relying on a single indicator.

**K value screening for the group with an available light meal canteen**. The group with a light meal canteen used light meal service satisfaction items as clustering variables, and the clustering performance was examined by traversing K = 2 to K = 8. The core indicators and sample distribution are shown in Table 17.

**Table 17:** Comparison of Clustering Indicators of Different K Values for Groups with a light meal cateen.

K Value	Silhouette Coefficient	Calinski-Harabasz Index	Cluster Distribution
2	0.196	35.69	50:94
3	0.143	30.28	26:66:52
4	0.131	26.97	10:39:43:52
5	0.126	23.77	27:31:27:53:6
6	0.138	21.34	28:22:17:52:6:19
7	0.113	20.30	29:17:16:33:4:18:27
8	0.107	18.09	19:15:16:30:23:7:26:8

The results of traversing the full gradient of K values show that when K = 2, the silhouette coefficient and CH index of the model were the highest among all parameter settings, indicating the best mathematical clustering performance. However, the sample sizes of the two clusters differed greatly, with a ratio of 50:94, meaning that the size of one group was nearly twice that of the other. The niche group had too few samples and weak representativeness, which could not support an effective interpretation of user heterogeneity, raising problems of ineffective clustering segmentation and one-sided results. When K increased to 3, although the silhouette coefficient and CH index declined slightly, the magnitude of the decline remained within a reasonable and controllable range, with no precipitous drop in clustering performance; meanwhile, the three groups were evenly distributed in size, with a ratio of 26:66:52, and there were no extremely small clusters or invalid segmented groups. In contrast, across the range K = 4 to K = 8, as the number of clusters increased, the evaluation indicators of the model continued to deteriorate, and the overall cluster discrimination and compactness declined steadily; moreover, extremely small clusters containing only single-digit numbers of samples appeared repeatedly, indicating severe fragmentation of the cluster structure and a lack of statistical representativeness and practical business interpretability. In summary, this study discarded K = 2, which had the best indicators but a severely imbalanced structure, and also rejected the invalid segmentation models at higher K values, ultimately determining K = 3 as the optimal number of clusters for the group with an available light meal canteen.

**K value screening for the group without a light meal canteen.** Similarly, a multi-K-value comparative screening was conducted for the group without a light meal canteen, and the core evaluation results are shown in Table 18.

**Table 18:** Comparison of Clustering Indicators of Different K Values for Groups without a light meal canteen.

K Value	Silhouette Coefficient	Calinski-Harabasz Index	Cluster Distribution
2	0.221	40.09	76:50
3	0.165	32.16	42:55:29
4	0.132	27.83	35:41:28:22
5	0.118	24.57	31:38:25:22:10
6	0.105	21.32	28:35:22:19:10:12
7	0.098	19.65	25:32:20:17:9:11:12
8	0.091	17.28	22:29:18:15:8:10:11:13

K = 2 was the globally optimal solution for the group without a light meal canteen, possessing the highest silhouette coefficient and CH index among all K values, with the best cluster compactness and discrimination. Meanwhile, the proportions of the two clusters were reasonable, with no extreme skew, and the group structure was stable, providing good statistical representativeness and business interpretability. Therefore, this study directly determined K = 2 as the optimal number of clusters for the group without a light meal canteen.

**(3) Clustering results and group profiling**

The silhouette coefficient for the group with an available light meal canteen was 0.143, and the CH index was 30.28. The cluster centroids of each group are shown in Table 19.

**Table 19:** Cluster‑centroid values for groups with a light meal canteen.

Cluster	Metric	Taste	Price	Freshness	Variety	Nutrition Label	Convenience
1	Importance	3.15	2.54	3.19	3.19	3.42	3.60
	Performance	2.92	3.00	3.15	2.73		2.85
2	Importance	4.42	4.39	4.59	4.45	4.38	4.45
	Performance	3.97	3.94	4.26	4.09		4.35
3	Importance	4.48	4.40	4.73	4.40	4.13	4.04
	Performance	3.15	2.94	3.38	3.06		3.25

The silhouette coefficient for the group without a light meal canteen was 0.221, and the CH index was 40.09. The cluster centroids of each group are shown in Table 20.

**Table 20:** Cluster‑centroid values for groups without a light meal canteen.

Indicator	Cluster 1	Cluster 2
Health Benefit	4.59	3.76
Taste Expectation	4.51	3.90
Price Willingness	3.64	2.88
Peer Influence	4.04	3.10
Campus Trend	4.26	3.12
Trust Standard	3.75	2.98
Convenience Access	3.88	3.02
Weight Control	4.08	3.12
Willingness Try	4.36	3.16
Frequency Increase	4.24	2.82
Recommend	3.93	2.70

Principal component analysis (PCA) was used to reduce the dimensionality of the clustering results for visualization, as shown in Figs 4 and 5, and the cluster centroids were plotted as radar charts, as shown in Figs 6 and 7. On this basis, the groups were named and their characteristics described, as shown in Table 21.

**Table 21:** Naming and core characteristics of college student light meal consumer segments.

Group	Name	Characteristics	Sample proportion
Groups with a light meal canteen	High‑satisfaction loyal users (Cluster1)	Predominantly female, mostly sophomores and juniors with moderate monthly living expenses. Nearly one‑third have fat‑reduction or low‑sugar dietary needs; they are fully satisfied with light‑food services including taste and ingredient freshness, showing consistent attitudes.	45.8% (66)
	Differentiated rational users (Cluster2)	Balanced gender ratio, mostly juniors and seniors with low‑to‑moderate monthly living expenses; only a small share have special dietary needs. They recognize ingredient freshness yet are dissatisfied with dish variety, with differentiated attitudes toward existing services.	36.1% (52)
	Low‑satisfaction wait‑and‑see users (Cluster3)	Predominantly male, with a high proportion of postgraduates and low monthly living expenses. Nearly half have special dietary needs; they give low evaluations on light‑food prices and dish variety and hold negative attitudes.	18.1% (26)
Groups without a light meal canteen	High‑demand expectant users (Cluster1)	Predominantly female, mostly upper‑grade undergraduates with moderate monthly living expenses. More than half have fat‑reduction or stomach‑care dietary needs; they recognize the health value of light food, are susceptible to social influences, and show strong willingness to try.	60.3% (76)
	Low‑demand wait‑and‑see users (Cluster2)	Balanced gender ratio with scattered grade distribution and low monthly living expenses; only a small share have special dietary needs. They hold neutral attitudes toward light food, are little affected by social factors, and have weak willingness to try.	39.7% (50)

**Fig 4:**
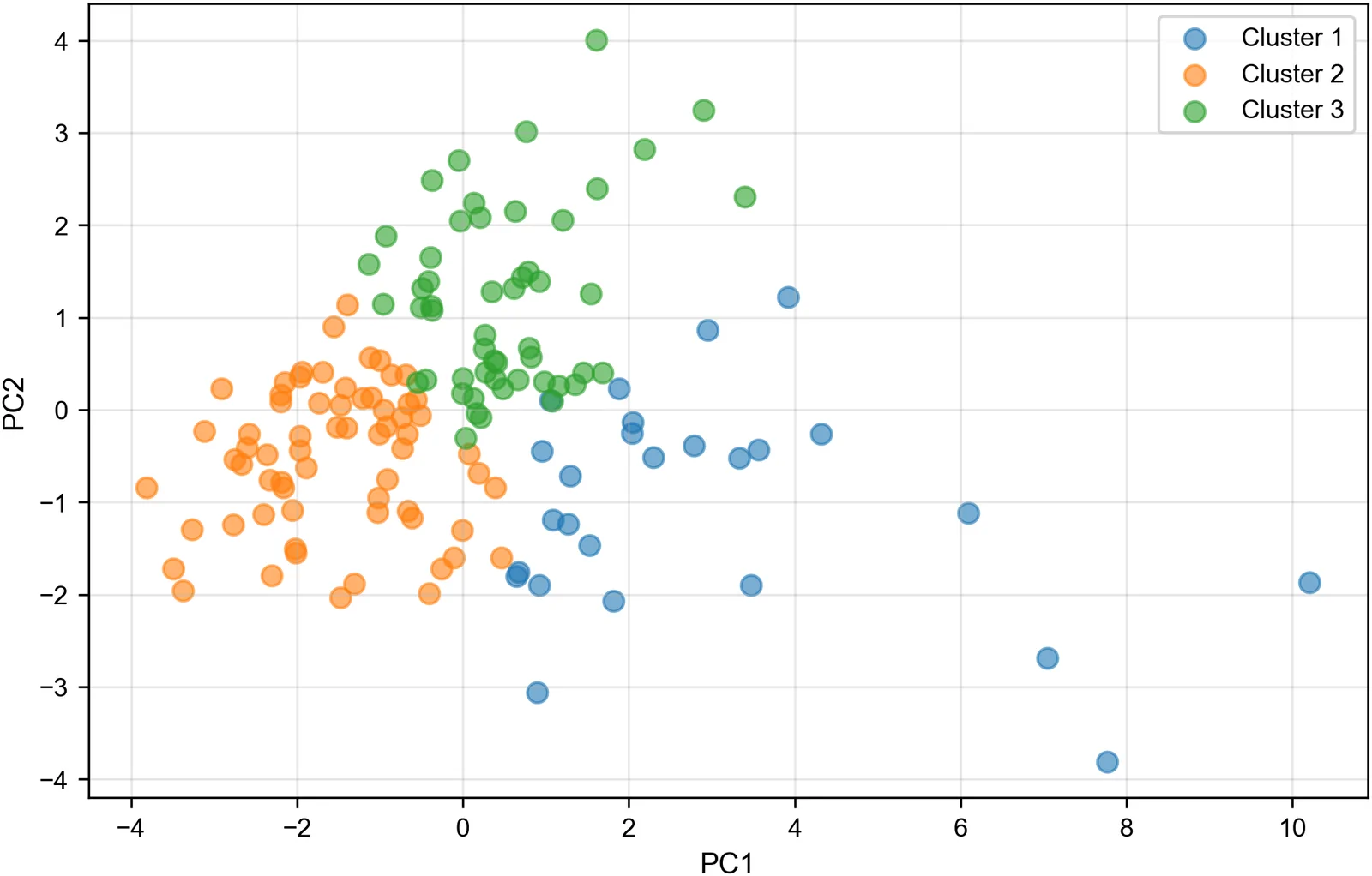
Clustering Results With Light Meal Canteen.

**Fig 5:**
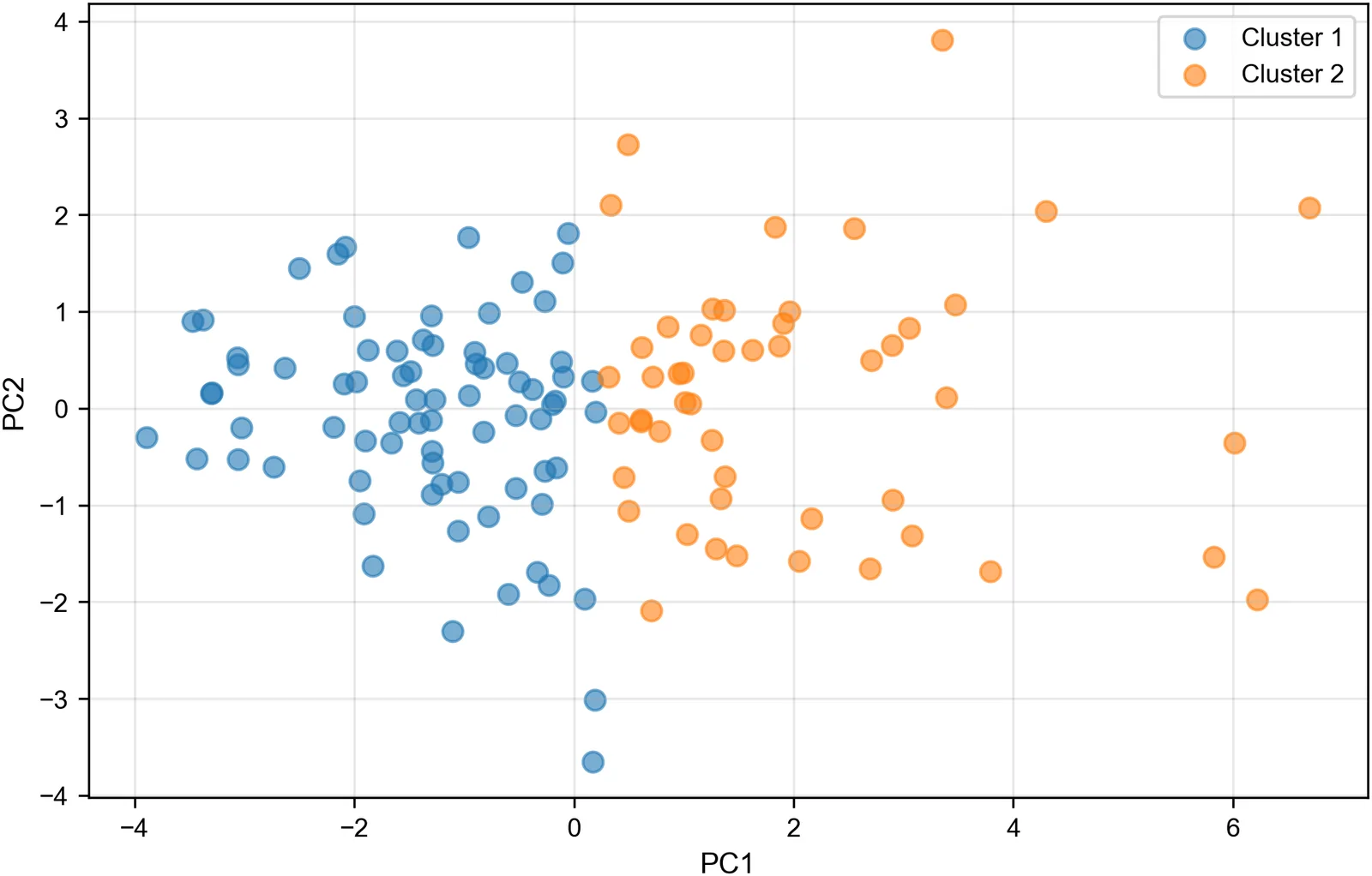
Clustering Results Without Light Meal Canteen.

**Fig 6:**
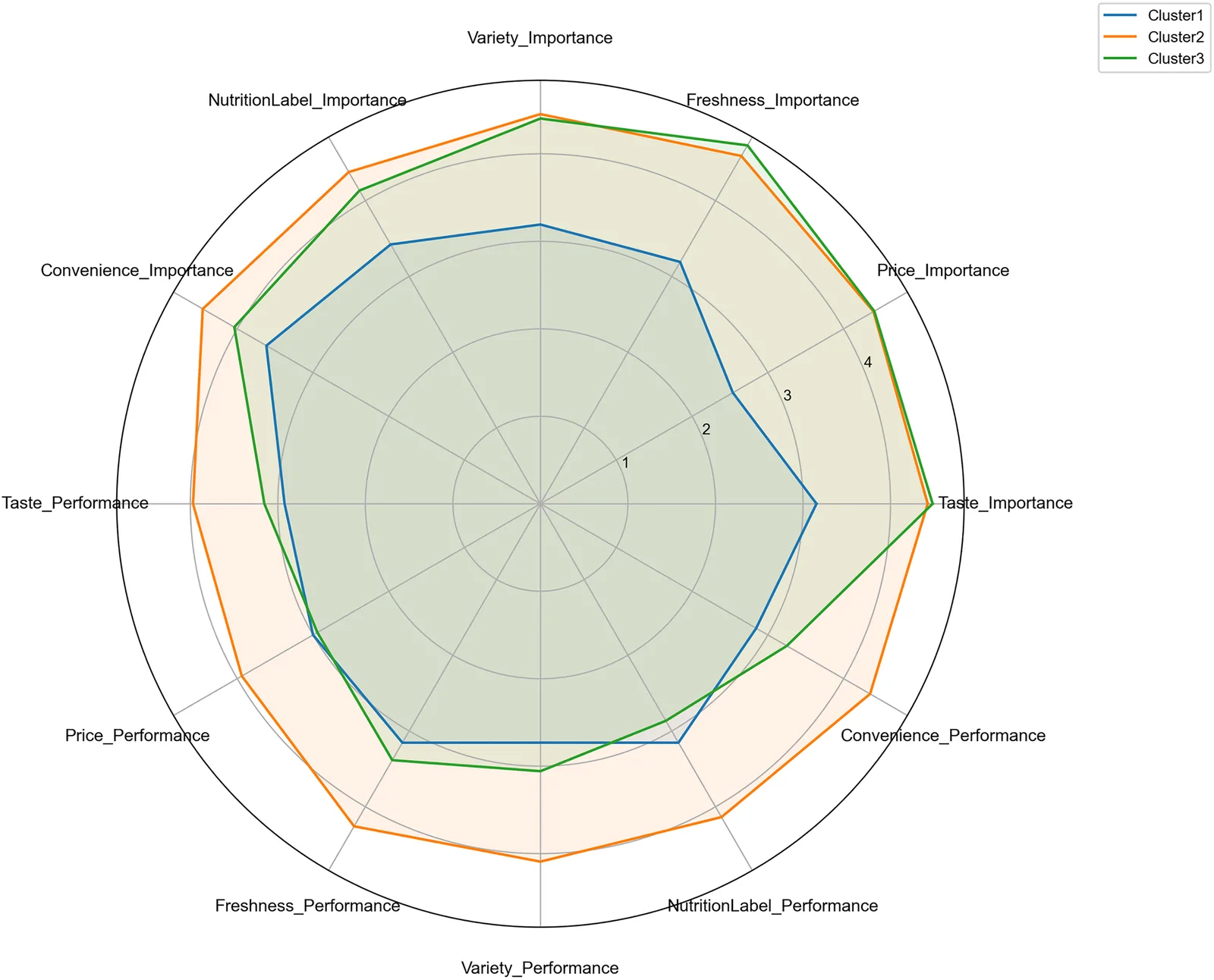
Radar Chart With Light Meal Canteen.

**Fig 7:**
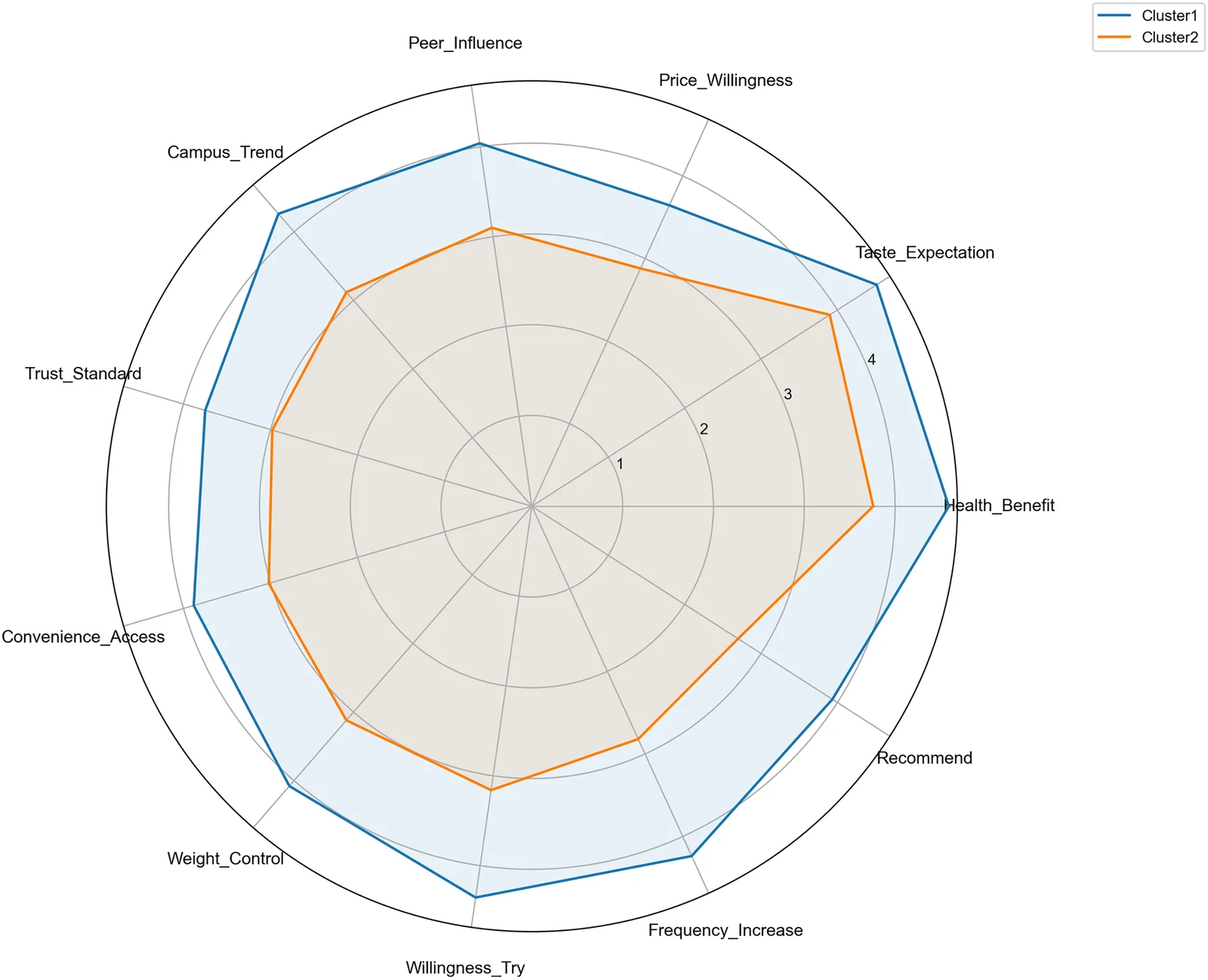
Radar Chart Without Light Meal Canteen.

## 5. Conclusions

Based on 270 valid questionnaires from seven major geographic regions of China and using a combination of multiple ordinal logistic regression, structural equation modeling, importance–performance analysis (IPA), and K-means clustering, this study draws the following conclusions:

(1) Regarding demand characteristics, college students’ light meal consumption exhibits four major features: diversified demands, regular-meal-oriented consumption timing, rigid price sensitivity, and functionalized product categories. Specific dietary needs are the core factor driving increased consumption frequency, whereas external supply conditions and demographic characteristics show no significant effects.
(2) Regarding the intention mechanism, in universities without a light meal supply, students’ consumption intention follows a multiple mediation pathway of “attitude–norm–intention,” with subjective norm showing the strongest direct effect, highlighting the key role of peer effects.
(3) Regarding service diagnosis, universities with a light meal supply generally exhibit supply–demand gaps across all dimensions, with taste and variety being the core shortcomings, while nutritional labeling and value for money represent low-cost entry points for optimization.
(4) Regarding group segmentation, significant heterogeneity exists within the student population: three groups can be identified in the group with an available light meal canteen—high-satisfaction loyal users, differentiated rational users, and low-satisfaction wait-and-see users—while two groups can be identified in the group without a light meal canteen—high-demand expectant users and low-demand wait-and-see users.
(5) Regarding practical implications, the provision of light meals in universities should be upgraded from merely adding a food stall to building an ecosystem: universities without supply should prioritize activating demand before proceeding with provision, while universities with supply should focus on taste and variety as the core areas for improvement and implement stratified operations.

## 6. Discussion

(1) Intr**insic health demands are the core driving force**

This study found that specific dietary needs were the only significant factor driving consumption frequency, whereas monthly living expenses and supply conditions were not significant. Previous studies have pointed out that willingness to pay and price factors are often regarded as the strongest predictors of healthy food purchase intention [13]; however, this study shows that in the relatively price-controllable setting of university canteens, price constraints give way to intrinsic health motivation. This is consistent with the core logic of the theory of planned behavior: behavioral attitude originates from individuals’ belief-based evaluation of behavioral outcomes [25], and demands such as fat reduction and stomach care constitute students’ clear expectations of health benefits, whose effect exceeds that of external conditions. A survey by China Youth Daily likewise showed that over 80% of college students have fat-reduction needs and nearly 60% have paid for light meals [6], corroborating the dominant role of intrinsic motivation. Therefore, the starting point for light meal provision in universities should be to activate health demands rather than merely adding food stalls.

**(2) The key role of subjective norm**

The structural equation model showed that attitude indirectly affects intention through subjective norm and perceived behavioral control, with subjective norm exhibiting the strongest direct effect. This is consistent with existing evidence that referent group norms have a significant influence on college students’ healthy eating intention—a study of campus café settings likewise revealed a disconnect between health preferences and consumption behavior [22]—while this study further demonstrates that in universities without supply, where students lack consumption experience, intention formation relies more on social information cues, and peer choices become a key reference compensating for the lack of experience. This suggests that before supply is implemented, a normative atmosphere for healthy eating should first be cultivated to lower the cognitive threshold for intention formation.

**(3) Taste and variety are the core shortcomings**

IPA analysis showed that taste and flavor, together with variety of dish types, constituted the areas for improvement, consistent with findings in campus foodservice quality research that taste and diversity are often identified as key shortcomings [23]. Drzal et al. systematically reviewed the barriers to and facilitators of healthy eating among college students, finding that the tension between taste preferences and health intentions is a key factor constraining healthy eating behavior [31]. A systematic review by Toc et al. further revealed that the mismatch between the accessibility and attractiveness of healthy options in the campus food environment is a core issue affecting students’ dietary patterns [32]. This study further reveals that in the light meal context, the tension of “healthy but not tasty” is amplified into the primary bottleneck constraining repeat purchases. Although previous studies have developed quantitative assessment tools for oil and salt perception [23], they have not answered the question of how to optimize. This study shows that the direction of optimization should not be simply increasing variety, but rather achieving functional stratification, replacing category accumulation with functional segmentation.

**(4) Group heterogeneity and stratified operations**

K-means clustering showed that three groups could be identified in the group with an available light meal canteen—high-satisfaction loyal users, differentiated rational users, and low-satisfaction wait-and-see users—while two groups could be identified in the group without a light meal canteen—high-demand expectant users and low-demand wait-and-see users. Previous studies have mostly treated college students as a homogeneous whole, or presented only basic consumption willingness and price preferences [12,13], lacking insight into segmented groups [14]. This study addresses this limitation and provides an empirical basis for stratified strategies: for the group with an available light meal canteen, variety should be expanded for differentiated rational users and the threshold for trying should be lowered for low-satisfaction wait-and-see users; for the group without a light meal canteen, high-demand expectant users should be treated as seed users to avoid resource misallocation caused by supply preceding demand.

## 7. Limitations

(1) **The generalizability of the sampling approach is limited**. This study adopted a combination of quota sampling and convenience sampling. Although basic coverage across the seven major geographic regions was achieved, the distribution of the sample with respect to institutional tier and disciplinary category may still be biased, and the unconditional generalizability of the conclusions to the entire population of Chinese college students is therefore limited.
(2) **The data rely on self-reports.** Consumption frequency and behavioral intention were both subjective measures and may be subject to social desirability bias; future research could incorporate objective data such as canteen consumption records for cross-validation.
(3) **The cross-sectional design makes it difficult to capture dynamic evolution.** This study could not observe the transformation of students’ intentions and behaviors before and after the implementation of supply; subsequent research could adopt a longitudinal design to reveal the mechanisms of behavioral change during the transition from a no-supply to a with-supply context.
(4) **There is still room for expansion in the selection of variables.** This study did not include potential moderating variables such as taste preferences, dietary culture, and institutional type; future research could further enrich the model specification to enhance explanatory power.**7. Theoretical and Practical Implications**

Theoretically, this study extends the classical theory of planned behavior framework to the context of campus light meal consumption, verifies the antecedent influence paths of attitude on subjective norm and perceived behavioral control, and reveals the multiple mediation mechanism of “attitude–norm/control–intention,” thereby providing a theoretical supplement to healthy food consumption research in a specific segmented context. At the same time, this study integrates ordinal logistic regression, SEM, IPA, and K-means clustering within a single analytical framework, achieving a full-chain characterization from identification of influencing factors to mechanism testing and further to service diagnosis and market segmentation, and providing a replicable methodological combination for campus foodservice research.

Practically, this study provides actionable strategic recommendations for the precise layout and refined optimization of light meal services in university canteens. For universities without supply, health education and norm cultivation should be prioritized to activate demand before proceeding with supply layout; for universities with supply, taste and variety should serve as the core areas for improvement, nutritional labeling and value for money should be used as low-cost entry points for optimization, and differentiated operations should be implemented for different groups. More importantly, the provision of light meals in universities should not be simply understood as adding a food stall, but should be regarded as a systematic project for building a healthy campus dietary ecosystem, in which demand cultivation, supply design, service optimization, and group operations must be advanced in a coordinated manner.

## Supporting information

S1 Data Data. (XLSX)
